# Clinicopathological features and prognostic nomogram of giant cell carcinoma of the lung: A population‐based study

**DOI:** 10.1111/crj.13586

**Published:** 2023-01-31

**Authors:** Jiang He, Jin‐Ping Ni, Guang‐Bin Li, Jie Yao, Bin Ni

**Affiliations:** ^1^ Department of Thoracic Surgery The First Affiliated Hospital of Soochow University Suzhou China; ^2^ Suzhou Kowloon Hospital Shanghai Jiaotong University School of Medicine Suzhou China

**Keywords:** giant cell carcinoma of the lung, nomogram, propensity score matching analysis, SEER database

## Abstract

**Background:**

Due to its rarity, the features and prognosis of giant cell carcinoma of the lung (GCCL) are not well defined. The present study aimed to describe the clinicopathological features and prognostic analysis of this rare disease, compare it with lung adenocarcinoma (LAC), further determine the prognostic factors and establish a nomogram.

**Methods:**

Patients diagnosed with GCCL and LAC were identified from the SEER database between 2004 and 2016. The features and survival between GCCL and LAC were compared in the unmatched and matched cohorts after propensity score matching (PSM) analysis. Univariate and multivariate Cox analyses were used to identify the prognostic factors, and a nomogram was constructed. Area under the curve (AUC), C‐index, calibration curve and decision curve analysis (DCA) were used to confirm the established nomogram.

**Results:**

A total of 295 patient diagnosed with GCCL and 149 082 patients with LAC were identified. Compared with LAC, patients with GCCL tend to be younger, male, black and have pathological Grade III/IV GCCL, more proportion of AJCC‐TNM‐IV, T3/T4 and distant metastases. The 1‐, 2‐ and 5‐year OS rates of the patients with GCCL were 21.7%, 13.4% and 7.9%, respectively. The median OS and CSS were 3 and 4 months, respectively. Patients with GCCL had significantly shorter OS and CSS than those with LAC in the unmatched and matched cohorts after PSM. Multivariate Cox analysis demonstrated that T, N and M stages and use of chemotherapy and surgery were independent of survival. Furthermore, we constructed a prognostic nomogram for OS and CSS by using independent prognostic factors. The C‐index of OS‐specific nomogram is 0.78 (0.74–0.81), and the C‐index of CSS‐specific nomogram is 0.77 (0.73–0.80). The calibration curve and ROC analysis showed good predictive capability of these nomograms. DCA showed that the nomogram had greater clinical practical value in predicting the OS and CSS of GCCL than TNM staging.

**Conclusion:**

GCCL have distinct clinicopathological characteristics and significantly worse clinical outcomes. Prognostic nomograms for overall survival (OS) and CSS were constructed.

## Graphical abstract

The 1‐, 2‐ and 5‐year overall survival rates of patients with giant cell carcinoma of the lung (GCCL) were 21.7%, 13.4% and 7.9%, respectively. GCCL patients had significantly worse survival than lung adenocarcinoma. The prognostic nomogram was constructed, which had greater clinical practical value in predicting the survival of GCCL patients than TNM staging system.

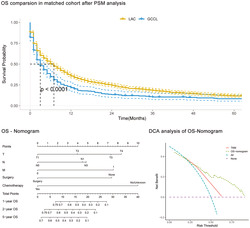

## 1 INTRODUCTION

Giant cell carcinoma of the lung (GCCL) is a rare subtype of lung cancer that has been categorized as pulmonary sarcomatoid carcinoma, along with pleomorphic carcinoma, spindle cell carcinoma, carcinosarcoma and pulmonary blastoma.^1^ Previous studies have shown that GCCL accounts for approximately 0.1%–0.4% of all lung cancers.^2^, ^3^ From a histopathological point of view, GCCL consists entirely of highly pleomorphic giant tumour cells, which can be easily distinguished from other common types of lung cancer, such as adenocarcinoma and squamous cell carcinoma.^4^ Patients with GCCL often present with non‐specific respiratory symptoms, including cough, haemoptysis, chest pain and dyspnoea, and these patients with symptoms are more likely to seek medical attention; however, some patients could also be asymptomatic similar to patients with other types of lung cancer.^4^, ^5^ Due to its rarity, previous studies on GCCL mainly focused on case reports or small sample cases series.^4^, ^6^, ^7^ According to previous sporadic reports, this rare disease is more prevalent in males and is associated with a history of smoking.^3^, ^8^, ^9^ Although GCCL can occur in any lung lobe, more than 50% of cases have lesions located in the upper lobes.^3^

Consistent with other non‐small cell lung cancer (NSCLC) subtypes, surgical resection remains the primary treatment option for GCCL. However, many patients have no chance of undergoing surgery because 60.3% of patients have metastasis at diagnosis.^3^ Thus, patients with GCCL were reported to have a very poor prognosis, with a 5‐year OS rate of only 16% with a median OS of 6 months, compared with 19% and 14.0 months in patients with non‐GCCL NSCLC.^3^ As for chemotherapy, previous retrospective studies and case reports suggested that GCCL is clinically aggressive with an inadequate response to antitumour chemotherapy, resulting in a very poor prognosis.^10^, ^11^ Vieira et al conducted a study with 97 patients with advanced lung sarcomatoid carcinomas, showing that only 16.5% of cases achieved partial response to chemotherapy, with median progression‐free survival and OS of 2.0 and 6.5 months.^11^ The efficacy of radiotherapy for the treatment of GCCL has not yet been established.

However, our understanding of GCCL often originates from case reports or single‐institutional case series owing to its rarity. More accurate information concerning the characteristics and clinical outcomes of this disease is needed in a larger cohort. Thus, we performed a retrospective, real‐world study on all patients with GCCL registered in the Surveillance, Epidemiology, and End Results (SEER) database from 2004 to 2016 to describe the clinicopathological characteristics of this rare disease and to determine potential prognostic factors.

## 2 MATERIALS AND METHODS

### 2.1 Patients

The SEER database from 18 population‐based cancer registries across the United States encompasses approximately 35% of the American population.^12^ The SEER‐18 database was selected to search for patients with GCCL diagnosed between 2004 and 2016. Data from each patient were extracted using the SEER*Stat software (Version 8.4.0). Diagnosed cases were identified using the specific codes of the International Classification of Diseases for Oncology, 3rd edition (ICD‐O), under which giant cell carcinoma (8031/3) is classified. Patients were included in the study if they met the following criteria: (1) one primary tumour only without multiple malignancies, (2) had complete data on their survival time and (3) had clear information on follow‐up. Patients diagnosed with lung adenocarcinoma (LAC) were also identified using ICD‐O‐3: 8140/3 under the same inclusion criteria to compare the characteristics and survival of patients with GCCL and adenocarcinoma. The publicly available nature of the SEER database qualified this study for ethical review exemption, and no informed consent was required.

### 2.2 Variable of interest

Variables, including age at diagnosis, sex, race, marital status, insurance status, primary site, differentiation grade, TNM stage, metastatic site, surgery, radiotherapy, chemotherapy, survival time, SEER cause‐specific death classification and vital status, were extracted from the SEER database. Primary outcome variables included OS and cancer‐specific survival (CSS). OS was defined as the interval from the time of diagnosis of GCCL to death caused by any reason or censored at the last known follow‐up, whereas CSS was defined as the interval from the time of diagnosis to death caused by GCCL or censored at the last known follow‐up.

### 2.3 Statistical analysis

The demographic and clinicopathological characteristics were summarized using descriptive statistics. Comparisons of categorical variables between GCCL and lung adenocarcinoma were conducted using the chi‐squared test. Kaplan–Meier curves and log‐rank tests were used to evaluate OS and CSS among the different groups. Propensity score matching (PSM) was used to balance the baseline characteristics between GCCL and lung adenocarcinoma when comparing OS and CSS between the two groups. Univariate and multivariate COX analyses were used to determine prognostic factors for OS and CSS in patients with GCCL. Meanwhile, we constructed a prognostic nomogram for predicting OS and CSS among patients, respectively. The area under the curve (AUC), C‐index and calibration curve were used to confirm the predictive accuracy and discriminability of the established nomogram. Decision curve analysis (DCA) was used to further confirm the clinical effectiveness of these nomograms. R software (Version 4.1.1; http://www.R-project.org) was used for statistical analyses. A two‐sided *P* value less than 0.05 was considered statistically significant.

## 3 RESULTS

### 3.1 Clinicopathological characteristics

In total, 295 patients diagnosed with GCCL and 149 082 with LAC were identified from the SEER database between 2004 and 2016. The baseline characteristics of the included patients are presented in Table 1. Comparative analysis showed that there were significant differences in age, sex, race, insurance status, pathological grade, TNM stage, T stage and M stage. Compared with LAC patients, GCCL patients tended to be younger than 60 years (37.3% vs. 25.1%), male (63.1% vs. 74.9%), black (17.3% vs. 12.3%) and pathological grade III/IV (49.5% vs. 26.2%). Regarding clinical stage, GCCL patients had a much higher proportion of AJCC‐TNM‐IV (63.1% vs. 56.1%), advanced T stage (T3/T4: 55.6% vs. 42.1%) and distant metastases (63.1% vs. 56.1%). There were no significant differences in the possibility of undergoing surgery, radiation or chemotherapy.

**TABLE 1:** Demographic and clinicopathological characteristics between GCCL and LAC

Characteristics	GCCL (*N* = 295)	LAC (*N* = 149 082)	*P* value
Age			
<60 years	110 (37.3%)	37 424 (25.1%)	<0.01
≥60 years	185 (62.7%)	111 658 (74.9%)	
Sex			
Female	109 (36.9%)	75 725 (50.8%)	<0.01
Male	186 (63.1%)	73 357 (49.2%)	
Race			
White	229 (77.6%)	116 238 (78.0%)	<0.01
Black	51 (17.3%)	18 333 (12.3%)	
Other	14 (4.7%)	14 151 (9.5%)	
Unknown	1 (0.3%)	360 (0.2%)	
Marital status			
Married	167 (56.6%)	76 512 (51.3%)	0.19
Unmarried	117 (39.7%)	66 139 (44.4%)	
Unknown	11 (3.7%)	6431 (4.3%)	
Insurance			
Insured	149 (50.5%)	97 632 (65.5%)	<0.01
Uninsured	14 (4.7%)	3851 (2.6%)	
Unknown	132 (44.7%)	47 599 (31.9%)	
Laterality			
Left	116 (39.3%)	56 156 (37.7%)	0.62
Right	160 (54.2%)	84 632 (56.8%)	
Unknown	19 (6.4%)	8294 (5.6%)	
Site			
Main bronchus	16 (5.4%)	4275 (2.9%)	0.12
Upper lobe	158 (53.6%)	76 703 (51.5%)	
Middle lobe	10 (3.4%)	6505 (4.4%)	
Lower lobe	64 (21.7%)	36 803 (24.7%)	
Overlapping lesion	3 (1.0%)	1586 (1.1%)	
Lung NOS	44 (14.9%)	23 210 (15.6%)	
Differentiated grade			
Grade I	0 (0%)	8472 (5.7%)	<0.01
Grade II	1 (0.3%)	27 101 (18.2%)	
Grade III	86 (29.2%)	38 110 (25.6%)	
Grade IV	60 (20.3%)	878 (0.6%)	
Unknown	148 (50.2%)	74 521 (50.0%)	
TNM			
I	25 (8.5%)	24 382 (16.4%)	<0.01
II	16 (5.4%)	7307 (4.9%)	
III	54 (18.3%)	27 090 (18.2%)	
IV	186 (63.1%)	83 702 (56.1%)	
Unknown	14 (4.7%)	6601 (4.4%)	
T			
T0	2 (0.7%)	944 (0.6%)	<0.01
T1	19 (6.4%)	28 997 (19.5%)	
T2	80 (27.1%)	38 191 (25.6%)	
T3	51 (17.3%)	19 272 (12.9%)	
T4	113 (38.3%)	43 466 (29.2%)	
Unknown	30 (10.2%)	18 212 (12.2%)	
N			
N0	90 (30.5%)	52 897 (35.5%)	0.22
N1	30 (10.2%)	11 923 (8.0%)	
N2	100 (33.9%)	51 527 (34.6%)	
N3	49 (16.6%)	20 449 (13.7%)	
Unknown	26 (8.8%)	12 286 (8.2%)	
M			
M0	102 (34.6%)	62 201 (41.7%)	0.04
M1	186 (63.1%)	83 675 (56.1%)	
Unknown	7 (2.4%)	3206 (2.2%)	
Radiation			
None/unknown	173 (58.6%)	91 491 (61.4%)	0.34
Yes	122 (41.4%)	57 591 (38.6%)	
Surgery			
None	228 (77.3%)	116 737 (78.3%)	0.32
Surgery	67 (22.7%)	31 409 (21.1%)	
Unknown	0 (0%)	936 (0.6%)	
Chemotherapy			
No/unknown	170 (57.6%)	79 082 (53.0%)	0.12
Yes	125 (42.4%)	70 000 (47.0%)	

### 3.2 Survival analysis

**FIGURE 1:**
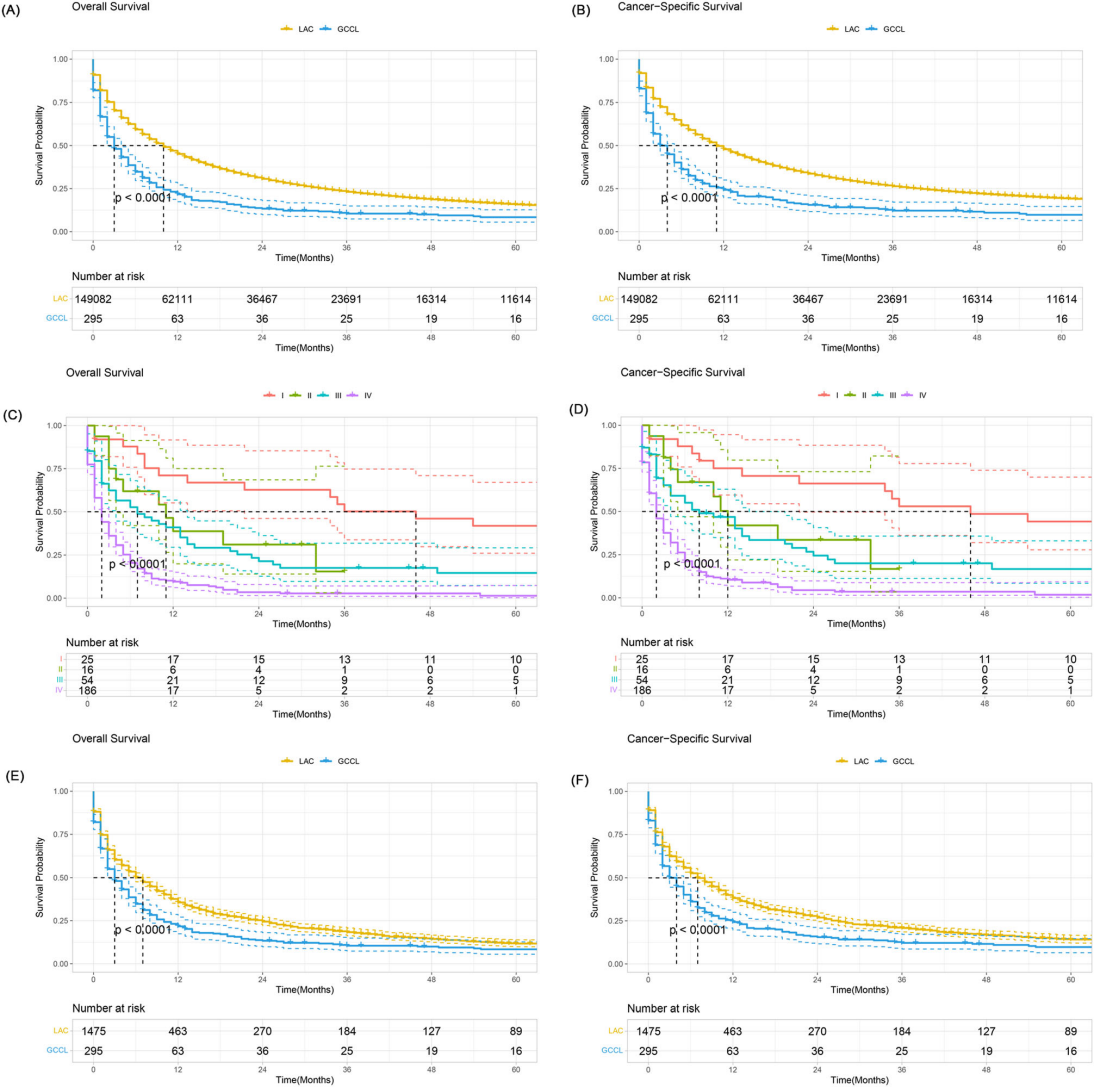
Kaplan–Meier analysis comparing overall survival and cancer‐specific survival between patients with GCCL and LAC in the entire cohort (A,B) and in the matched cohort (E,F). Overall survival and cancer‐specific survival among GCCL patients with different AJCC‐TNM staging (C,D)

Kaplan–Meier analysis showed that the OS and CSS of patients with GCCL were significantly worse than those of patients with LAC (Figure 1A, B). The 1‐, 2‐ and 5‐year OS rates of patients with GCCL were 21.7%, 13.4% and 7.9%, respectively, whereas the 1‐, 2‐ and 5‐year CSS of GCCL patients were 24.3%, 15.6% and 9.2%, respectively. The median OS and CSS were 3 and 4 months, respectively. In the LAC group, the 1‐, 2‐ and 5‐year OS rates were 45.1%, 30.7% and 15.8%, respectively, whereas the 1‐, 2‐ and 5‐year CSS rates were 47.9%, 33.7% and 19.4%, respectively. The median OS and CSS times were 10 and 11 months, respectively. The OS and CSS of GCCL patients stratified by TNM stage were present in Figure 1C,D.

To balance the baseline characteristics between GCCL and LAC patients, we performed a PSM analysis (1:5) to obtain a matched cohort of 295 patients and 1475 LAC patients with LAC. There was no significant difference between the GCCL and LAC patients in the matched cohort (*P* > 0.05 for all; Table 2). The survival analysis in the matched cohort also demonstrated that patients with GCCL had significantly shorter OS and CSS than those with LAC (Figure 1E,F). In the LAC group of the matched cohort, the 1‐, 2‐ and 5‐year OS rates were 35.6%, 24.5% and 11.7%, respectively, whereas the 1‐, 2‐ and 5‐year CSS rates were 38.2%, 26.9% and 14.1%, respectively. The median OS and CSS of these LAC patients in the matched cohort were 7 and 7 months, respectively.

**TABLE 2:** Demographic and clinicopathological characteristics between GCCL and LAC in the matched cohort (1:5)

Characteristics	GCCL (*N* = 295)	LAC (*N* = 1475)	*P* value
Age			
<60 years	110 (37.3%)	520 (35.3%)	0.55
≥60 years	185 (62.7%)	955 (64.7%)	
Sex			
Female	109 (36.9%)	536 (36.3%)	0.90
Male	186 (63.1%)	939 (63.7%)	
Race			
White	229 (77.6%)	1158 (78.5%)	0.98
Black	51 (17.3%)	247 (16.7%)	
Other	14 (4.7%)	64 (4.3%)	
Unknown	1 (0.3%)	6 (0.4%)	
Marital status			
Married	167 (56.6%)	853 (57.8%)	0.92
Unmarried	117 (39.7%)	570 (38.6%)	
Unknown	11 (3.7%)	52 (3.5%)	
Insurance			
Insured	149 (50.5%)	772 (52.3%)	0.84
Uninsured	14 (4.7%)	70 (4.7%)	
Unknown	132 (44.7%)	633 (42.9%)	
Laterality			
Left	116 (39.3%)	582 (39.5%)	0.86
Right	160 (54.2%)	794 (53.8%)	
Unknown	19 (6.4%)	99 (6.7%)	
Site			
Main bronchus	16 (5.4%)	67 (4.5%)	0.98
Upper lobe	158 (53.6%)	807 (54.7%)	
Middle lobe	10 (3.4%)	42 (2.8%)	
Lower lobe	64 (21.7%)	322 (21.85)	
Overlapping lesion	3 (1.0%)	14 (0.9%)	
Lung NOS	44 (14.9%)	223 (15.1%)	
Differentiated grade			
Grade I	0 (0%)	0 (0%)	0.88
Grade II	1 (0.3%)	8 (0.5%)	
Grade III	86 (29.2%)	456 (30.9%)	
Grade IV	60 (20.3%)	302 (20.5%)	
Unknown	148 (50.2%)	709 (48.1%)	
TNM			
I	25 (8.5%)	128 (8.7%)	0.99
II	16 (5.4%)	85 (5.8%)	
III	54 (18.3%)	287 (19.5%)	
IV	186 (63.1%)	909 (61.6%)	
Unknown	14 (4.7%)	66 (4.5%)	
T			
T0	2 (0.7%)	9 (0.6%)	0.98
T1	19 (6.4%)	81 (5.5%)	
T2	80 (27.1%)	411 (27.9%)	
T3	51 (17.3%)	253 (17.2%)	
T4	113 (38.3%)	582 (39.5%)	
Unknown	30 (10.2%)	139 (9.4%)	
N			
N0	90 (30.5%)	404 (27.4%)	0.69
N1	30 (10.2%)	167 (11.3%)	
N2	100 (33.9%)	517 (35.1%)	
N3	49 (16.6%)	275 (18.6%)	
Unknown	26 (8.8%)	112 (7.6%)	
M			
M0	102 (34.6%)	538 (36.5%)	0.74
M1	186 (63.1%)	909 (61.6%)	
Unknown	7 (2.4%)	28 (1.9%)	
Radiation			
None/unknown	173 (58.6%)	849 (57.6%)	0.78
Yes	122 (41.4%)	626 (42.4%)	
Surgery			
None	228 (77.3%)	1119 (75.9%)	0.65
Surgery	67 (22.7%)	356 (24.1)	
Unknown	0 (0%)	0 (0%)	
Chemotherapy			
No/unknown	170 (57.6%)	818 (55.5%)	0.54
Yes	125 (42.4%)	657 (44.5)	

### 3.3 Prognostic factor for OS and CSS among GCCL patients

Afterwards, we used univariate and multivariate COX analyses to identify the prognostic factors for OS and CSS among patients with GCCL. As shown in Table 3, the multivariate Cox analysis demonstrated that T, N and M stages and the use of chemotherapy and surgery were independent of OS (*P* < 0.05 for all). As shown in Table 4, the multivariate Cox analysis demonstrated that T stage, N stage, M stage and the use of chemotherapy and surgery were independent of CSS (*P* < 0.05 for all).

**TABLE 3:** Univariate and multivariate Cox analysis for OS among GCCL patients

Characteristics	Univariate Cox analysis		Multivariate Cox analysis	
	HR with 95% CI	*P* value	HR with 95% CI	*P* value
Age				
<60 years	Reference		Reference	
≥60 years	1.18 (0.92–1.52)	0.38		
Sex				
Female	Reference		Reference	
Male	1.08 (0.84–1.39)	0.67		
Race				
White	Reference		Reference	
Black	1.42 (1.04–1.95)	0.03	1.02 (0.71–1.47)	0.90
Other	1.07 (0.60–1.91)	0.82	0.93 (0.50–1.74)	0.82
Marital status				
Married	Reference			
Unmarried	1.27 (0.99–1.63)	0.06		
Insurance				
Insured	Reference			
Uninsured	1.40 (0.79–2.48)	0.25		
Laterality				
Left	Reference			
Right	0.96 (0.75–1.24)	0.78		
Site				
Main bronchus	Reference		Reference	
Upper lobe	0.55 (0.33–0.93)	0.02	0.83 (0.46–1.50)	0.54
Middle lobe	0.56 (0.24–1.32)	0.19	0.92 (0.36–2.39)	0.87
Lower lobe	0.55 (0.31–0.95)	0.03	0.99 (0.52–1.88)	0.98
Overlapping lesion	0.15 (0.03–0.64)	0.01	0.52 (0.11–2.44)	0.41
Lung NOS	1.06 (0.59–1.88)	0.85	1.40 (0.70–2.78)	0.34
Differentiated grade				
Grade II/III	Reference		Reference	
Grade IV	1.09 (0.77–1.55)	0.63		
T				
T1	Reference		Reference	
T2	1.42 (0.84–2.43)	0.19	1.64 (0.93–2.91)	0.09
T3	2.09 (1.19–3.66)	0.01	2.18 (1.17–4.07)	0.01
T4	2.60 (1.55–4.36)	<0.01	2.26 (1.27–4.04)	0.01
N				
N0	Reference		Reference	
N1	1.51 (0.97–2.36)	0.07	1.58 (0.97–2.56)	0.06
N2	1.74 (1.28–2.38)	<0.01	1.49 (1.02–2.16)	0.04
N3	2.11 (1.44–3.08)	<0.01	1.45 (0.92–2.27)	0.11
M				
M0	Reference		Reference	
M1	2.78 (2.09–3.70)	<0.01	1.99 (1.41–2.81)	<0.01
Radiation				
None/unknown	Reference			
Yes	0.91 (0.71–1.17)	0.47		
Surgery				
None	Reference		Reference	
Surgery	0.30 (0.21–0.42)	<0.01	0.47 (0.30–0.74)	<0.01
Chemotherapy				
No/unknown	Reference		Reference	
Yes	0.60 (0.47–0.77)	<0.01	0.40 (0.29–0.54)	<0.01

**TABLE 4:** Univariate and multivariate Cox analysis for CSS among GCCL patients

Characteristics	Univariate Cox analysis		Multivariate Cox analysis	
	HR with 95% CI	*P* value	HR with 95% CI	HR with 95% CI
Age				
<60 years	Reference		Reference	
≥60 years	1.22 (0.94–1.59)	0.13		
Sex				
Female	Reference		Reference	
Male	1.04 (0.80–1.35)	0.78		
Race				
White	Reference		Reference	
Black	1.41 (1.02–1.96)	0.04	1.03 (0.71–1.50)	0.87
Other	1.07 (0.58–1.97)	0.82	0.95 (0.50–1.84)	0.89
Marital status				
Married	Reference		Reference	
Unmarried	1.29 (0.99–1.67)	0.06		
Insurance				
Insured	Reference		Reference	
Uninsured	1.36 (0.75–2.47)	0.31		
Laterality				
Left	Reference		Reference	
Right	0.99 (0.76–1.29)	0.96		
Site				
Main bronchus	Reference		Reference	
Upper lobe	0.54 (0.32–0.93)	0.03	0.83 (0.45–1.52)	0.54
Middle lobe	0.52 (0.21–1.29)	0.16	0.86 (0.31–2.38)	0.78
Lower lobe	0.57 (0.32–1.01)	0.05	1.07 (0.55–2.07)	0.84
Overlapping lesion	0.09 (0.01–0.66)	0.02	0.33 (0.04–2.65)	0.30
Lung NOS	1.07 (0.59–1.93)	0.83	1.42 (0.70–2.90)	0.33
Differentiated grade				
Grade II/III	Reference		Reference	
Grade IV	1.05 (0.73–1.51)	0.77		
Unknown	1.16 (0.87–1.55)	0.32		
T				
T1	Reference		Reference	
T2	1.73 (0.95–3.14)	0.07	2.00 (1.06–3.75)	0.03
T3	2.36 (1.26–4.42)	0.01	2.50 (1.26–4.97)	0.01
T4	3.07 (1.72–5.50)	<0.01	2.79 (1.47–5.30)	<0.01
N				
N0	Reference		Reference	
N1	1.54 (0.97–2.43)	0.07	1.52 (0.93–2.50)	0.09
N2	1.76 (1.28–2.43)	<0.01	1.42 (0.97–2.08)	0.07
N3	2.02 (1.36–3.01)	<0.01	1.30 (0.81–2.07)	0.27
M				
M0	Reference		Reference	
M1	2.99 (2.22–4.04)	<0.01	2.26 (1.57–3.25)	<0.01
Radiation				
None/unknown	Reference		Reference	
Yes	0.93 (0.72–1.20)	0.58		
Surgery				
None	Reference		Reference	
Surgery	0.30 (0.21–0.43)	<0.01	0.51 (0.32–0.81)	<0.01
Chemotherapy				
No/unknown	Reference		Reference	
Yes	0.61 (0.47–0.79)	<0.01	0.42 (0.31–0.58)	<0.01

### 3.4 Prognostic nomogram for OS and CSS

**FIGURE 2:**
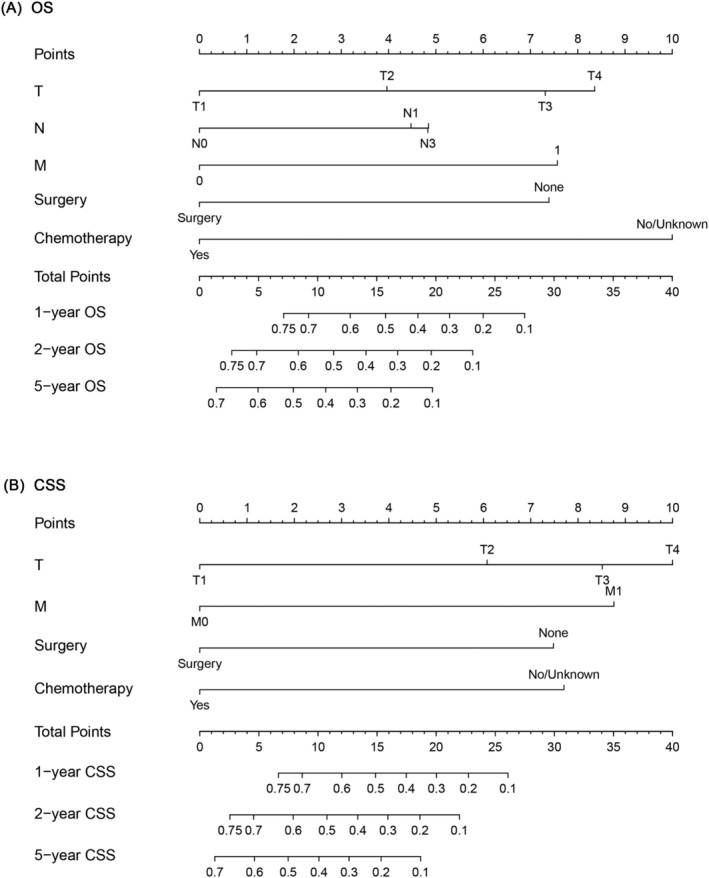
Nomogram for predicting the OS (A) and CSS (B) of patients with GCCL

**FIGURE 3:**
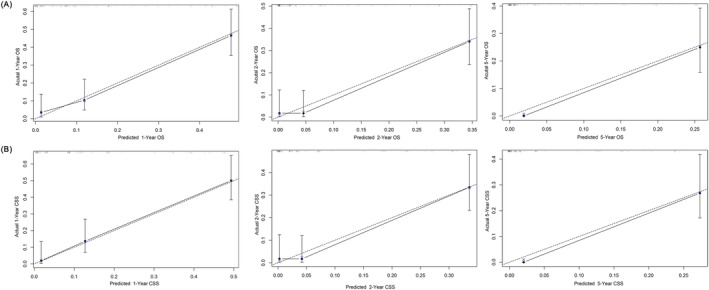
Calibration curves of the OS‐/CSS‐specific nomograms. Calibration curves of 1‐, 2‐ and 5‐year OS (A) and CSS (B) for patients with GCCL

**FIGURE 4:**
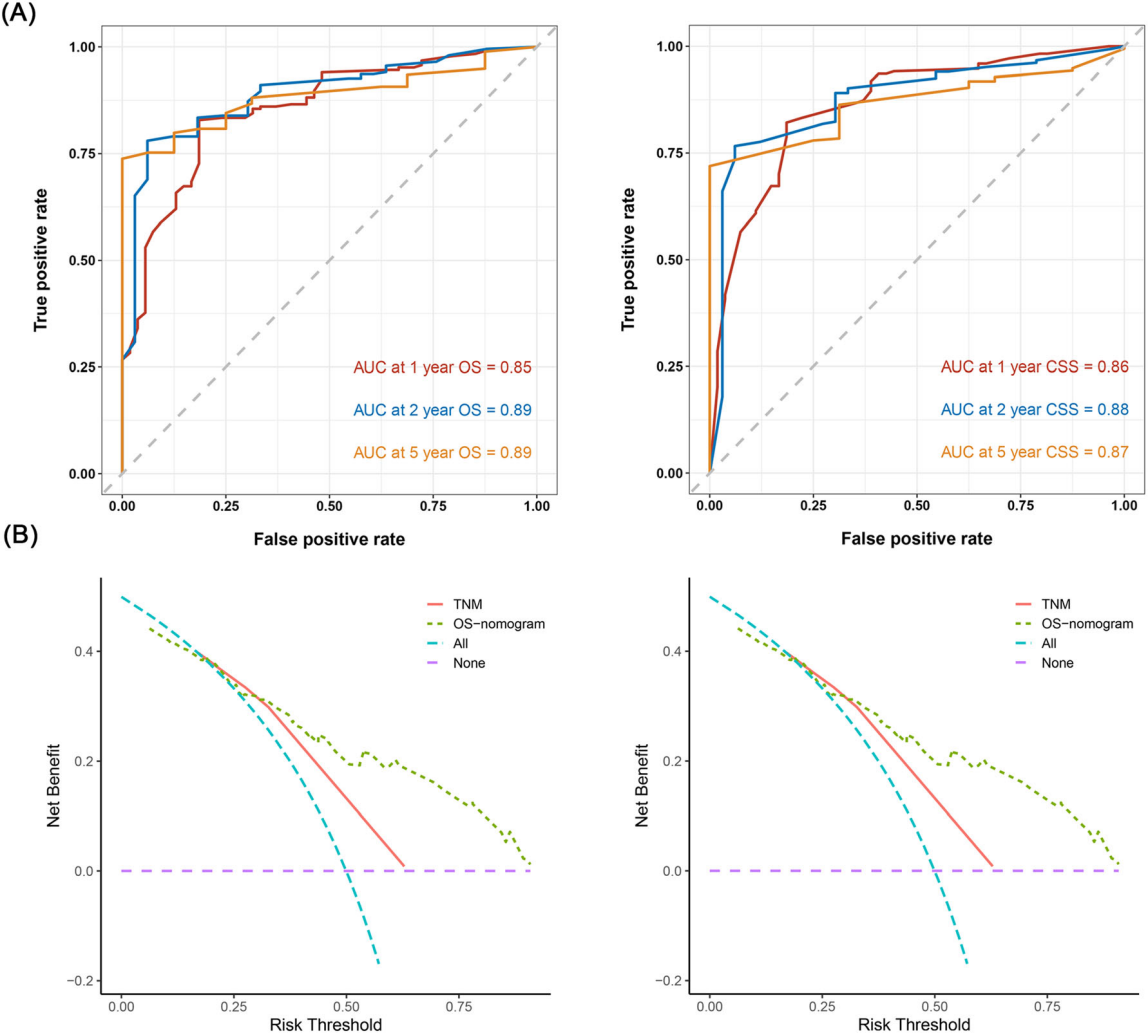
ROC curves analysis and DCA of the OS‐/CSS‐specific nomogram. ROC curves of the ability of nomogram to predict 1‐, 2‐ and 5‐year OS and CSS (A). DCA of the nomogram and TNM stage for OS and CSS prediction of patients with GCCL (B)

Furthermore, we constructed a prognostic nomogram for OS and CSS using independent prognostic factors from multivariate Cox analysis. As shown in Figure 2A, chemotherapy contributed the most to OS, followed by T stage, M stage, surgery and N stage, and the C‐index of the OS‐specific nomogram was 0.78 (0.74–0.81). As for the CSS‐specific nomogram (Figure 2B), the T stage contributed the most to CSS, followed by the M stage, chemotherapy and surgery. The C‐index value of the CSS‐specific nomogram was 0.77 (0.7–0.80). The calibration curve showed good predictive capability of the nomogram (Figure 3). ROC analysis of the nomogram revealed that the AUC of 1‐, 2‐ and 5‐year OS was 0.853, 0.889 and 0.885, respectively, whereas the AUC of 1‐, 2‐ and 5‐year CSS was 0.862, 0.876 and 0.866, respectively (Figure 4A). DCA was used to evaluate the clinical value of the OS/CSS‐specific nomogram. As shown in Figure 4B, the nomogram demonstrated a significant positive net benefit from the risk of all‐cause death and cancer‐related death and was better than the traditional TNM staging system, indicating its great clinical practical value in predicting the OS and CSS of GCCL.

## 4 DISCUSSION

Existing literature on primary GCCL is limited; therefore, its characteristics and survival have not been well defined. In the present study, we compared the clinicopathological features and prognosis of patients with primary GCCL and LAC, the most common type of NSCLC, in unmatched and matched cohorts. Meanwhile, we identified independent prognostic factors for OS and CSS and constructed a prognostic nomogram for predicting survival. We used the AUC, C‐index, calibration curve and DCA to confirm the predictive accuracy and discriminability of the established nomogram and its clinical effectiveness. To the best of our knowledge, this is the first retrospective study to establish a nomogram to predict the prognosis of patients with GCCL using data from the SEER database.

Owing to the rarity of GCCL, the paucity of studies describing its clinicopathological characteristics necessitated a SEER database‐based retrospective study. The demographic analysis in this study showed that 63.1% of the GCCL patients were male with a relatively younger age, which is similar to that reported previously. Wang et al reported that 83.3% of patients with GCCL were male and 41.7% were younger than 60 years in a real‐world small sample cohort of 12 GCCL patients.^3^ Several studies have reported that GCCL often had a history of smoking, which could not be described due to lack of smoking information in the SEER database. As a primary site, GCCL could occur in any lung lobe, similar to other NSCLC, but previous studies have reported that it is observed much more frequently in the upper lobes. In our cohort, more than half of the patients with GCCL had upper lobe lesions, but there was no significant difference between patients with GCCL and LAC. Similarly, GCCL is a poorly differentiated tumour with a high possibility of metastases through lymph and blood circulation.^9^ Almost half (49.5) of GCCL patients were diagnosed with poorly differentiated (Grade III) and undifferentiated (Grade IV) disease, with 60.7% and 63.1% of patients developing lymph node metastases and distant metastases, respectively, at initial diagnosis, all of which were significantly higher than those of LAC patients. In addition, the number of GCCL patients with insurance was lower than that of LAC patients between 2004 and 2016, which may be a potential reason for GCCL patients with advanced tumour stage. However, this finding should be validated in future studies.

Similar to other types of NSCLC, surgical resection is an effective treatment that provides adequate local control for patients with GCCL.^13^ However, most patients are at quite advanced disease at the initial diagnosis and lose the chance to undergo surgery. Even after surgery, most patients with GCCL experience tumour recurrence or death within 16–18 months; thus, radiation and chemotherapy have also been used to treat GCCL. Unfortunately, several previous studies have suggested that GCCL was not respond well to chemotherapy, which is inconsistent with our data. In our cohort, patients undergoing chemotherapy had significantly better survival rates than those who did not receive chemotherapy. Multivariate Cox analysis showed that chemotherapy was an independent prognostic factor for both OS and CSS. In established nomograms, chemotherapy contributed the most to both OS and CSS among all the prognostic factors. To date, no investigations have shown the efficacy of radiation in the treatment of GCCL. Our data are the first to report the association between the use of radiation and survival, and no significant differences between patients with and without radiation could be observed. Due to poor survival and poor response to conventional treatment, an increasing number of treatment options, such as molecular‐targeted therapy, have been attempted in patients with GCCL. GCCL was reported to have a lower rate of sensitive EGFR mutations than other types of NSCLC, and the efficacy of EGFR‐targeted therapy for GCCL has not been well elucidated.^14^, ^15^ A previous study showed that patients with GCCL with EGFR mutations may not obtain adequate clinical benefit from EGFR‐targeted therapy. Wang reported that only two out of four patients with GCCL carrying EGFR mutations benefited from EGFR‐TKIs.^3^ Previous studies have demonstrated that EGFR mutational heterogeneity in PSC may cause resistance to EGFR‐TKIs.^16^ Another study also demonstrated that the presence of a targetable mutation in GCCL was correlated with the presence of morphological or immunohistochemical adenocarcinomatous differentiation.^17^ In addition, Hsieb et al found lung adenocarcinoma with sarcomatoid transformation after EGFR‐TKI treatment and chemotherapy and six patients demonstrated giant cell features with aberrant MET activation and PD‐L1 expression after EGFR‐TKI treatment.^18^ Thus, as a subtype of pulmonary PSC, MET alteration may be a potential treatment target. Although the SEER database lacks information on gene mutations, knowledge concerning targeted therapy needs to be updated in the future. In addition, previous studies reported high PD‐L1 expression in some patients with PSC, suggesting the promise of immunotherapy in patients with GCCL.^19^, ^20^

Of course, there are several limitations that should be acknowledged because this was a retrospective study based on the SEER database. First, although we only included patients diagnosed between 2004 and 2016, there is also a lack of information on the variable, such as 4.7% patients having no information on TNM, almost half having no information on pathological grade, etc. The unknown information may affect the results of survival analysis based on multivariate Cox analysis. For example, N stage was only identified to be significantly associated with CSS among GCCL patients in univariate Cox analysis, but not multivariate Cox analysis. Second, some important characteristics affecting prognosis were not included in the SEER database, such as molecular gene mutations, the use of targeted therapy, immunotherapy, recurrence and treatment response. Third, owing to the rarity of this disease, we could not validate our established nomogram in the external cohort. Thus, future investigations are required to confirm the accuracy and reliability of the established nomogram.

In conclusion, we conducted a retrospective study to describe the features and prognosis of GCCL using data from the SEER database. GCCL have distinct clinicopathological characteristics and significantly worse clinical outcomes. Moreover, prognostic factors were determined using multivariate Cox regression analysis, resulting in the establishment of an OS‐/CSS‐specific nomogram, which enables physicians to more accurately predict the survival of patients with GCCL.

## AUTHOR CONTRIBUTIONS

Conception and design: Jiang He and Bin Ni. Administrative support: Bin Ni. Provision of study materials or patients: Jiang He, Jin‐Ping Ni. Collection and assembly of data: Jiang He, Jin‐Ping Ni, Guang‐Bin Li. Data analysis and interpretation: Jiang He, Jin‐Ping Ni, Jie Yao, Bin Ni. Manuscript writing: All authors. Final approval of manuscript: All authors.

## CONFLICT OF INTEREST STATEMENT

All authors declare no conflict of interests.

## ETHICS APPROVAL STATEMENT

Institutional Review Board approval was not required since the data were de‐identified for previous study.

## Data Availability

The data that support the findings of this study are available from the corresponding author upon reasonable request.
